# Constitutive TSH receptor activation as a hallmark of thyroid autonomy

**DOI:** 10.1007/s12020-020-02270-z

**Published:** 2020-04-17

**Authors:** Dagmar Führer

**Affiliations:** Department of Endocrinology, Diabetes and Metabolism, University Hospital Essen, University Duisburg-Essen, Hufelandstr. 55, 45177 Essen, Germany

**Keywords:** TSH receptor, Somatic and germline mutation, Toxic adenoma, Toxic multinodular goitre, Hereditary non-autoimmune hyperthyroidism, Inverse agonist and antagonists

## Abstract

Since its cloning more than 30 years ago, the thyrotropin receptor (TSHR) has emerged as a pivotal player in thyroid physiology and pathophysiology. In particular, hyperthyroidism due to autoimmune disease or thyroid autonomy is linked with TSHR activation via autoantibodies or mutations respectively. This review summarises clinical aspects of constitutive TSH receptor activation by naturally occurring somatic or germline TSHR mutations resulting in TSH-independent thyroid function and cell proliferation.

## Constitutive TSH receptor activation as a new principle in endocrine tumours and a driver of thyroid autonomy

The group of Dumont and Vassart were among the first to suggest that any molecular lesion leading to constitutive activation of the cAMP cascade could be responsible for the growth and functional properties of autonomous thyroid nodules [1]. In support of this, it was shown that transgenic mice with thyroid expression of the adenosine A2 receptor mimic the phenotype of thyroid autonomy in humans [2]. In the first and pivotal study by the Brussels lab in 1993, 9 out of 11 toxic thyroid nodules harboured an activating TSHR mutation [3]. Subsequent studies comprising larger sample series showed that TSHR mutations are not only present in up to 82% of solitary toxic nodules [4–11] but also in autonomous nodules within toxic multinodular goitres [12–14]. The majority of these mutations were localised in the TSHR transmembrane domain and only rarely in the extracellular domain [15]. All TSHR mutations were confined to clonal autonomous tissue (=somatic mutations) and were heterozygous in line with a gain-of-function mutation exerting a dominant effect [16]. Furthermore, using archival tissues of euthyroid goitres from an iodine deficient area, somatic TSHR mutations were identified in microscopic areas with high 125-I labelling indicating autonomous tissues on autoradiography [17]. This finding illustrates that gain-of-function TSHR mutations are implicated in the early steps of thyroid autonomy. In parallel, Gs-alpha mutations (gsp) which likewise confer constitutive cAMP activation were detected in 5–30% of toxic thyroid nodules, that did not harbour a TSHR mutation, sustaining the initial hypothesis that alterations of several proteins may indeed contribute to constitutive activation of the cAMP pathway as a hallmark of thyroid autonomy.

## Lessons from in vitro characterisation of TSH receptor mutations

Functional characterisation of the identified TSHR mutations has mostly been performed in COS-7 cells and has demonstrated constitutive adenylylcylase activation, in addition to activation of phospholipase C-protein kinase C signalling by some mutations [3, 4, 18]. Already early on, different magnitudes of functional activity became apparent for the distinct gain-of-function TSHR mutations. Moreover, in vitro studies showed that cell surface expression of the TSHR mutants was reduced compared with the wild-type receptor either due to decreased processing of the mutant TSHR protein or alternatively increased mutant turn-over by internalisation. Importantly, this is not an artificial in vitro phenomenon, since reduced TSHR expression was also demonstrated ex vivo by immunhistochemical analysis of thyroid tissue from patients with gain-of-function TSHR mutations compared with normal and Graves’ disease thyroid tissue [19]. Detailed functional analysis of naturally occurring TSHR mutants and subsequent extensive modelling studies by several groups over the past 20 years has provided important insights into the mechanism of TSHR activation, embedded in general concepts of G protein coupled receptor (GPCR) function [20]. One new concept that emerged from these mechanistic studies was the idea that small molecules could be developed that act as inverse agonists or antagonists against, e.g. antibody driven TSH receptor activation in Graves’ disease and ophthalmopathy [21, 22]. As another example, it was recently demonstrated that the TSHR can also form complexes with other non-GPCR membrane proteins such as the mono-carboxylate transporter 8, which expressed on the basolateral membrane of thyrocytes is involved in thyroid hormone release [23]. This hints at truly complex regulation of thyroid hormone production at the level of the thyroid gland and takes the thinking from an individual receptor to the broader and hitherto understudied consideration of interacting protein networks in the thyrocyte membrane, which may be relevant for a better understanding of thyroid disease.

## Understanding the biological consequences of TSHR mutations for thyroid tumorigenesis

Distinct biological properties of various TSHR mutations and gsp were subsequently demonstrated in rat thyroid follicular cells and human thyrocytes [24, 25]. The major finding of these studies conducted in Marian Ludgate’s lab was that the behaviour of TSHR mutations in the thyroid context was not identical to in vitro analysis of the same mutations in non-thyroidal COS-7 cells. While induction of TSH-independent thyroid function was confirmed for all investigated TSHR mutants, TSH-independent thyroid cell proliferation was only observed in some mutations [24]. This clearly demonstrated the importance of the cellular context for a true understanding of the pathogenic contribution of TSHR activation to thyroid autonomy. Subsequent analyses, including proteomic studies in my lab underlined that even though signalling properties of gain-of-function TSHR are similar (with respect to the cAMP cascade), they are not identical and include involvement of other non-PKA pathways, at least in rat FRTL-5 cells [26]. These finding might also explain differences in cell proliferation capacity observed for the distinct activating TSHR mutations in thyroid cells. Furthermore, using mutant TSHR stably expressing FRTL-5 cells as an in vitro model of thyroid autonomy, we demonstrated that exposure to iodine in early phases of thyroid autonomy downregulated transcription of genes involved in cell proliferation [27, 28]. This finding is in line with epidemiological observations from the Pescopagano study [29], suggesting that improved iodine supply may actually hamper progression of early stage thyroid autonomy.

However, the precise mechanism that drive the evolution from thyrocytes harbouring a gain-of-function TSHR mutation to a clinically apparent toxic thyroid nodule are still far from being understood, adding to the ongoing discussion that additional alterations may be required. In this context, a second hit mutation was identified in enhancer of zeste homologue 1 (EZH1) in toxic thyroid nodules by whole-exome sequencing [30]. This mutation occurred in the catalytic subunit of the polycomb repressive complex 2 of EZH1, which is involved in embryonic stem cell pluripotency and plasticity and has been linked to cancer aggressiveness. Functional characterisation in rat thyroid cells showed that this EZH1 mutation confers increased histone H3 trimethylation and promotes cell proliferation. Interestingly, EZH1 mutations were found in 27% of 123 toxic nodules, and only in tumours, which also harboured a somatic gain-of-function TSHR mutation. This novel finding notwithstanding, not all toxic adenomas display an EZH1 mutation and from screening of other benign and malignant thyroid tumours it appears that EZH1 mutations at least in codon 571 are not principally involved in regulation of proliferation in these tumours [30]. In the future further mechanistic insights into the development of macroscopic thyroid autonomy may be derived from the first gain-of-function TSHR mutant mouse model that has only very recently been established [31].

## Clinical impact of constitutive TSHR activation in the rare condition of hereditary non-autoimmune hyperthyroidism

**Fig. 1:**
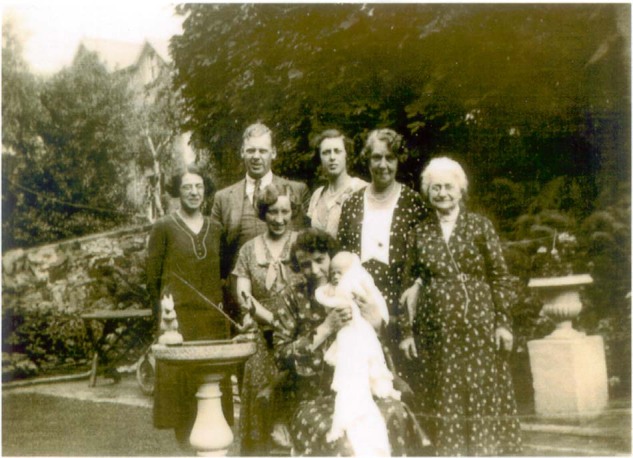
Familial non-autoimmune hyperthyroidism. This photo from the 1930s shows several members of the Welsh kindred with familial non-autoimmune hyperthyroidism. The old lady on the right side is the great-great-great grandmother of the index patient. The baby in the front row is the grandmother of the index patient, who was also found to harbour a gain-of-function TSHR germline mutation in Marian Ludgate’s lab (with kind permission of the family)

Constitutively activating TSHR mutations may also occur as germline mutations and in the situation cause hereditary non-autoimmune hyperthyroidism that is transmitted in an autosomal dominant manner [5, 32]. The clinical impact of germline TSH receptor activation is exemplified by the Welsh kindred described by the Ludgate lab in 2000 [33]. In this family, hyperthyroidism and goitre were prevalent with high frequency over three generations with putative involvement of at least three further generations now deceased. The laboratory finding of borderline thyroid autoantibodies (thyroglobulin, thyroperoxidase antibodies) in some of the affected family members led to the diagnosis of familial occurrence of Graves’ disease though absence of thyroid eye disease and relapse of hyperthyroidism after thyroid surgery were noted. Still the initial presentation of hyperthyroidism in conjunction with thyrotoxicosis in the—at that time 4-year-old offspring—was not regarded as highly unusual, since Graves’ disease accounts for most cases of thyrotoxicosis in childhood. However, when TSHR antibodies were determined in the child and were found to be absent, further investigation of the family was pursued. This led to the identification of a novel TSHR mutation localised in codon 463 of the second membrane-spanning region. The mutation was present in all family members with non-autoimmune hyperthyroidism and was absent in members with no clinical evidence for thyroid disease. Functional analysis of the mutation confirmed its gain-of-function nature and showed functional characteristics in agreement with previously studied TSHR mutations. The identification of this gain-of-function germline mutation determined the necessity for thyroid ablation in all affected members to prevent relapses of thyrotoxicosis. Moreover, molecular analysis offered the possibility for screening, allowing for early diagnosis in three young cousins of the index patient that were living in Asia. Finally, once hereditary disease is suspected, determination of the disease aetiology is mandatory because of the medical and legal requirement for genetic counselling of the patients, their relatives and offspring (Fig. 1).

## Conclusion

In sum, constitutive activation of the cAMP cascade, predominantly via TSHR mutations, is the molecular hallmark of thyroid autonomy in its various presentation as solitary toxic adenoma, toxic multinodular goitre or even hereditary non-autoimmune hyperthyroidism [34]. The identification of gain-of-function TSHR mutations in these conditions has largely substantiated the clinical management of thyroid autonomy by thyroid ablation (surgery or radioiodine) since spontaneous remission will neither occur in clonal lesions harbouring a TSHR mutation nor in individuals with gain-of-function TSHR germline mutations. Many open questions on TSHR function and thyroid disease remain to be resolved and this journey will be ongoing with development of small molecules as a first example and further discoveries facilitated by novel methodologies and advances in animal models and stem cell research including the first generation of mouse and human thyroid organoids [35, 36].
